# Mechanism of Zhen Wu Decoction in the Treatment of Heart Failure Based on Network Pharmacology and Molecular Docking

**DOI:** 10.1155/2022/4877920

**Published:** 2022-03-15

**Authors:** Chen-Yu Ma, Yu-Qian Ma, Min Deng

**Affiliations:** ^1^Cixi Third People's Hospital, Zhejiang, China; ^2^Wenzhou Medical University, Wenzhou, China; ^3^Emergency Medicine Department, Zhejiang Hospital of Integrated Traditional Chinese and Western Medicine, Hangzhou, China

## Abstract

Heart failure (HF) is a serious manifestation or advanced stage of various cardiovascular diseases, and its mortality and rehospitalization rate are still on the rise in China. Based on the network pharmacology method, 59 components of Zhen Wu decoction (ZWD) and 83 target genes related to HF were obtained. Through the PPI network, four potential therapeutic targets were identified: AKT1, IL6, JUN, and MAPK8. The beneficial components of ZWD might intervene HF through the AGE-RAGE signalling pathway in the diabetes component, fluid shear stress and atherosclerosis, the TNF signalling pathway, TB, and Kaposi sarcoma related herpesvirus infection, according to a KEGG enrichment study. The protein interaction network of candidate targets was constructed by the STRING database, and the protein interaction network was clustered by MEODE software. GO and KEGG enrichment analyses were performed on the core modules obtained by clustering. Finally, AutoDock Vina software was used for molecular docking verification of key targets and active ingredients. The result was that 75 active ingredients and 109 genes were screened as potential active ingredients and potential targets of Shengjie Tongyu decoction for CHF treatment. The main active components were quercetin, luteolin, kaempferol, dehydrated icariin, isorhamnetin, formononetin, and other flavonoids. Il-6, MAPK1, MAPK8, AKT1, VEGFA, and JUN were selected as the core targets. Molecular docking showed that the key components were well connected with the target. GO enrichment analysis showed that Shengjie Tongyu decoction could play a role through multiple biological pathways including angiogenesis, regulation of endothelial cell proliferation, binding of cytokine receptors, negative regulation of apoptotic signalling pathways, regulation of nitric oxide synthase activity, and reactive oxygen metabolism. Key pathways mainly focus on the toll-like receptor signalling pathway, nod-like receptor signalling pathway, MAPK signalling pathway, mTOR signalling pathway, JAK-STAT signalling pathway, VEGF signalling pathway, and other pathways. Through molecular docking technology, it was found that a variety of effective components in ZWD, such as kaempferol. Molecular docking technology has preliminatively verified the network pharmacology and laid a foundation for the follow-up pharmacological research.

## 1. Background

Heart failure (HF) refers to a variety of heart function or organic diseases caused by cardiac insufficiency or dysfunction, resulting in cardiac output which cannot meet the metabolic needs of the disease [1]. After the illness, organ deficiency, dyspnea, fluid retention, and so on seriously threaten the life safety of patients [2]. Heart failure (HF) is a prevalent kind of critical clinical disease. According to cardiac systolic/diastolic dysfunction, it can be divided into systolic HF and diastolic HF. Rendering to different clinical manifestations, it can be divided into left HF, right HF, and total HF [3]. In developed countries, the mortality rate is 1.5%–2.0%, and the pervasiveness proportion of individuals more than 70 years of age is more than or equal to 10%, while in China, the mortality rate of hospitalized patients is about 4.1% [4]. Primary myocardial damage and abnormality are the main causes of HF, and in addition to cardiovascular disease, noncardiovascular disease can also lead to it. At present, it is a chronic, spontaneous progressive disease [5]. According to the HF diagnosis and treatment quality evaluation and control index expert consensus 2021 in China, cardiac activation is the key factor that causes the development of HF. It is recommended to use the early use of sarkoba valsartan drugs to reduce the incidence rate and mortality [6]. Chinese medicine [7] is a comprehensive medical system that has been passed down for over two millennia.

Zhen Wu decoction (ZWD) comes from the treatise on febrile and miscellaneous diseases by Zhang Zhongjing. Modern research shows that it has a therapeutic effect on HF and other diseases [8]. Traditional Chinese medicine and its compounds have the characteristics of multicomponent, low selectivity, and multitarget interaction. This complexity makes it difficult to control the treatment; it lacks a reasonable and effective safety evaluation system and is relatively difficult to analyze its action and compatibility mechanism [9]. In recent years, network pharmacology has been rising in the world. It has the characteristics of multidirectional cross-fusion. It can realize the comprehensive network analysis of traditional Chinese medicine and its compound prescriptions and understand the pathogenesis of diseases from the system level. It has significant advantages and the potential to clarify the mechanism of action and explain the law of prescription composition [10, 11], because the integrity and systematicness of its research strategy correspond to the overall concept of traditional Chinese medicine and syndrome differentiation and treatment. As a result, one such study employs network pharmacology to investigate the pharmacodynamic material basis and molecular basis of Zhen Wu decoction in the therapies of HF, creates the “pharmacodynamic element goal continuum” relationship network, investigates the multicomponent, multitarget, and multichannel mechanism of Zhen Wu decoction, and preliminarily validates the results using docking study technology to provide the foundation for basic experimental research and clinical trials.

## 2. Data and Methods

### 2.1. Acquisition of Effective Components and Targets of Core Drugs

Input each medica of ZWD into TCMSP [12] data step by step (https://tcms-pw.com/tcmsp.php). Oral bioavailability is determined by the same principle [13] ≥ 30%, drug-like (DL) ≥ 0.18, was selected to obtain the effective components and target of core drugs. At the same time, the structure of the effective components was retrieved and saved in mol2 format. Import the abovementioned targets to the UniProt [14] website (https://www.UniProt.org/). Gene mapping is performed towards obtaining gene symbols to the corresponding target.

### 2.2. Acquisition of Disease-Drug Common Target Genes

In the human gene database, GeneCards (https://genecards.Weizmann.ac.il/v3/) and MalaCards (https://www.malecards.org/) were used. To obtain the target genes of coronary heart disease, we searched with the keyword “hyperlipidemia” in the abovementioned drug target genes, and disease target genes were introduced into the Venn diagram web (http://bioinformatics.PSB.ugent.be/webtools/Venn/). The common illness and therapeutic target genes were discovered.

### 2.3. Construction of Protein-Protein Interaction (PPI) Network for Key Targets

The suitable target genes of the disorders and medications mentioned above have been entered into the STRING website [15]. The species chosen as “*Homo sapiens*,” and the associated connections between protein targets were determined. Following the PPI file export, the graphs were improved with Cytoscape software, and the 10 leading essential genes (hub genes) in the PPI network were determined based on the degree values.

### 2.4. GO Enrichment Analysis and KEGG Enrichment Analysis

The illness medication commonality genes were added to the David database, and the species “*Homo sapiens*” was chosen for gene ontology (GO) and Kyoto Encyclopedia of Genes and Genomes (KEGG) signal pathway enrichment analyses [16, 17]. And the alleyway map of the signal pathways closely related to hypertension is obtained. The abovementioned results are imported into R software for visual operation of relevant results and data.

### 2.5. Composition-Target-Signal Pathway Network Diagram

Potential targets are imported into the David6.8 (HTTP://David, abcc. Ncifcrf. Gov/) gene function analysis tool. The identifier is selected. The list type were set to official gene symbol, gene list, and species is limited to Homo sapiens. Submit List is clicked for the GO function and KEGG enrichment analysis. At the same time, the ENSG encoding corresponding to potential genes is obtained by gene ID conversion. The whole human genome is taken as the background gene. The ENSG codes obtained were imported into the OmicShare (WWW. omicshare.com/tools) website for KEGG pathway annotation number analysis. To create the network design of active ingredient, target, and signal pathways, the active ingredient, target, and signal routes were loaded into the Cytoscape 3.6.0 programme.

### 2.6. Molecular Docking Verification

1. The PDB structure of hub protein was acquired by researching hub genes on the RCSB PDB database (https://www.rcsb.org/search). The abovementioned compounds were entered into the DS programme and prepared using the “prepare ligands” feature. To locate the probable active surface area in the protein cavities, remove water, ligand classes, and irrelevant side chains from the receptors and click on receptor-ligand interactions > define and edit the binding > from receptor cavities. (3) CDOCKER INTERACTION ENERGY (molecular docking binding energy) was utilised to identify and rank the optimum molecular conformations of every docking molecule. The findings reveal that the smaller the binding energy of the docking study conformation, the more stable the binding notion is and the more likely the reactive receptor molecule and ligand will bind.

## 3. Results

### 3.1. Active Ingredients and Targets of Core Drugs

From the TCMSP website, there are 13 active ingredients in Paeoniae Radix Alba, 7 active ingredients in Atractylodes macrocephala Koidz, 15 active ingredients in Poria Cocos (Schw.) Wolf, 21 active ingredients in Aconiti Lateralis Radix Praeparata, and 5 active ingredients in Zingiber officinale Roscoe, with 59 constituents (Table 1). The abovementioned effective components were input into the TCMSP website and searched for 85 effective component targets. Then, these target proteins were introduced into the UniProt website and transformed into a corresponding gene symbol.

### 3.2. Common Target Genes of ZWD and HF

**Figure 1:**
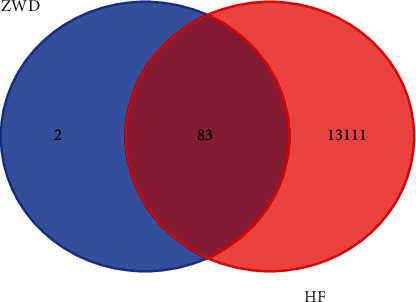
Common target genes of ZWD and HF.

13194 genes targets of hyperlipidemia were obtained since the GeneCards website, and disease and drug boards were imported to draw Venn diagram website for Wayne map analysis, and 83 common target genes of illness and drugs were obtained, as shown in Figure 1.

### 3.3. Key Target PPI Network

**Figure 2:**
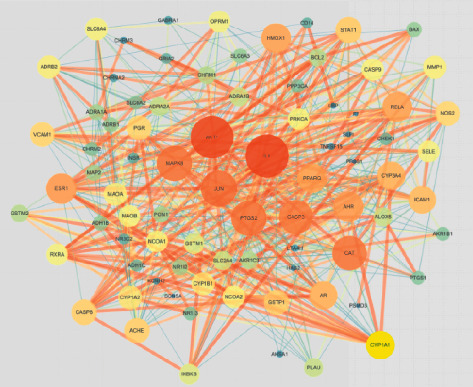
PPI network of common target genes of ZWD and HF.

**Figure 3:**
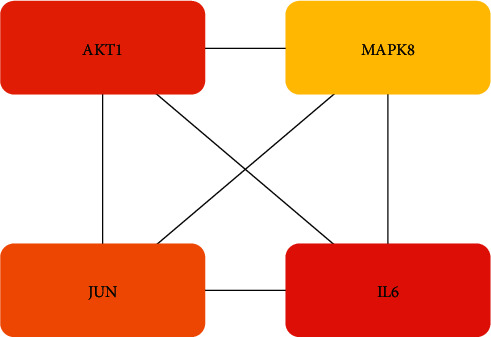
TOP4 genes of PPI network by degree value.

As shown in Figure 2, the PPI network map was created after the 83 frequent genes were added to the string database. Targets appear as nodes and are connected by edges in a PPI network. The more dense the edges of key targets are, the larger the nodes are, which means the more important role they play in the PPI network. The hub plug-in in the Cytoscape programme is used to retrieve the top 4 hub genes (core genes) according to the degree value after the PPI network is stored in TSV format, as shown in Figure 3.

### 3.4. KEGG Pathway Enrichment Analysis with GO Enrichment Analysis

**Figure 4:**
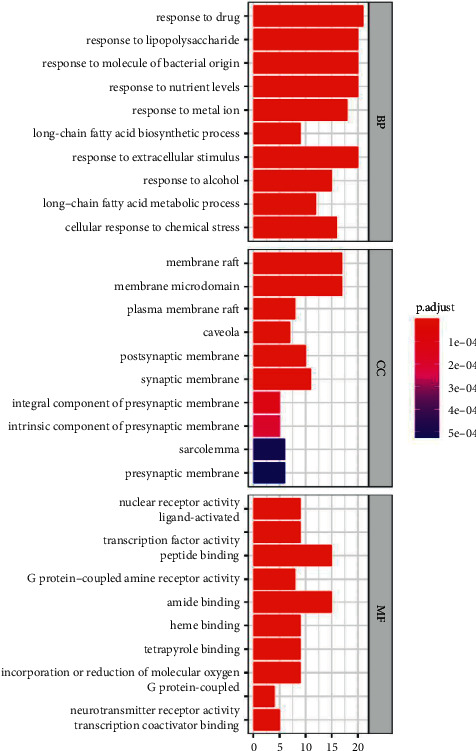
GO analysis of common target genes of ZWD and HF.

**Figure 5:**
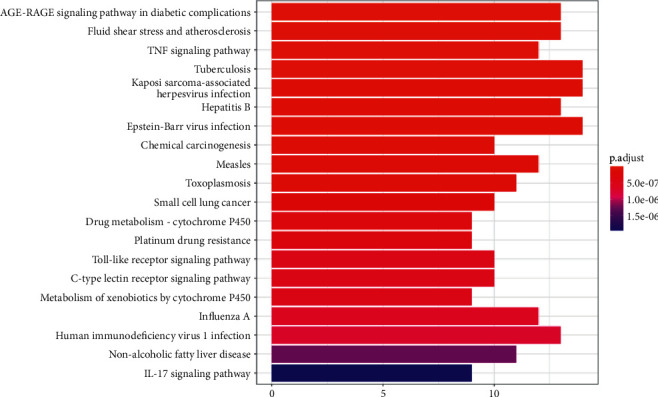
KEGG analysis of common target genes of ZWD and HF.

As shown in Figure 4, the 53 shared target genes of ZWD and HF were entered into the David database for ontologies (GO) enrichment research, which included three components: biological process (BP), cellular component (CC), and molecular function (MF). GO is mainly related to response to drugs, response to lipopolysaccharides, response to molecules of bacterial origin, and other BPs; membrane raft, micromembrane domain, plasma membrane raft, caveola, and other Mfs; nuclear receptor activity, live activated transcription factor activity, peptide binding, G protein-coupled amine receptor activity, and other MFs. KEGG enrichment analysis mainly involves the AGE-RAGE signalling pathway in diabetic composition, fluid shear stress and atherosclerosis, the TNF signalling pathway, tuberculosis, Kaposi sarcoma-associated herpesvirus infection, etc., as shown in Figure 5.

### 3.5. Component-Target-Pathway Network Analysis of ZWD

**Figure 6:**
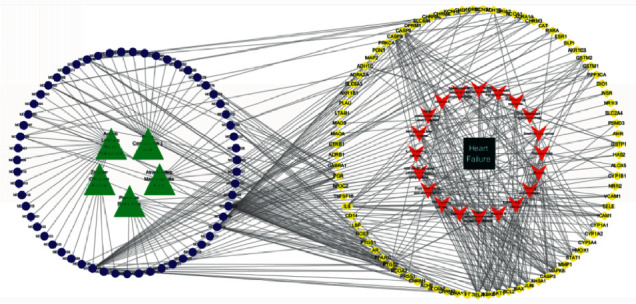
Network of component-targets pathways of ZWD treating HF.

To more clearly show the relationship between components, targets, and pathways of ZWD in the treatment of HF, the network of pathways, active ingredients, and core targets was constructed using Cytoscape software (Figure 6). A network graph contains nodes and edges. The green node represents drugs, the blue node represents active ingredients, the yellow node represents target genes, the red node represents signalling pathways, and the black node represents disease. The network diagram can directly show that ZWD own features of multicomponent, multitarget, and multichannel interaction in hyperlipidemia.

### 3.6. Molecular Docking Results

**Figure 7:**
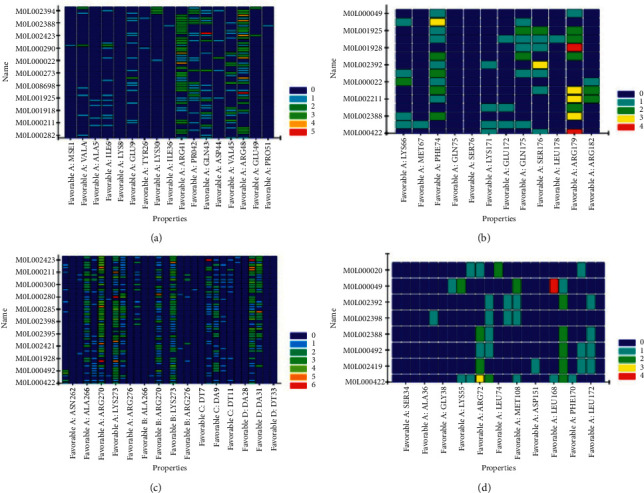
Docking heatmap of ligands and hub proteins.

**Figure 8:**
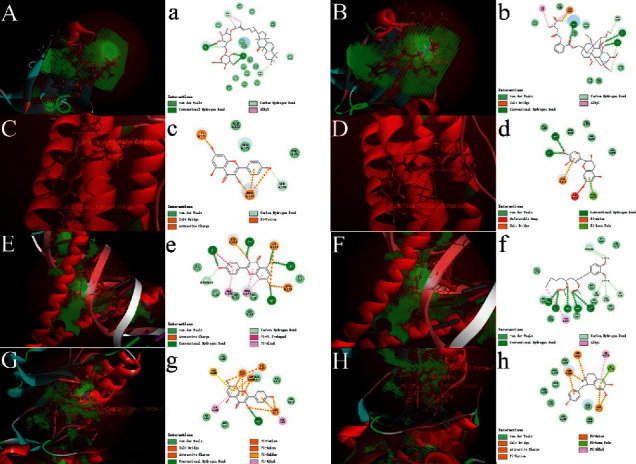
3(d) and 2D graph of docking conformations of ligands and hub proteins.

According to the method in 1.6 for molecular verification, the docking results of each group are shown by the heat map in Figure 7. The protein group is on the horizontal axis while the ligand is on the vertical axis. The redder the color, the stronger the binding force is. Each group's first two docking results with the highest energy score are shown as three-dimensional and two-dimensional graphs. The three-dimensional graph shows the spatial relationship between the ligand and the protein. The two-dimensional graph shows the chemical bond type formed by the ligand and the amino acid group or residue of the protein. The docking scores of the AKT1 group were MOL002433、MOL002394, 56.1364 and 53.2491, respectively, as shown in Figures 8(a) and 8(b). In the IL6 group, MOL 000422 and MOL 000492, the docking scores were 74.0031 and 48.4424, respectively, as shown in Figures 8(c) and 8(d) . In Jun group, MOL 000422 and MOL006129 were used, with docking scores of 75.7236 and 69.2787, as shown in Figures 8(e) and 8(f). In mapk8 group, MOL 000422 and MOL 002419, with docking scores of 48.9098 and 47.1798, as shown in Figures 8(g) and 8(h).

## 4. Discussion

HF is a range of complicated clinical syndromes, a significant manifestation or end phase of several heart illnesses [18]. It is produced by aberrant cardiac structure and/or function induced by various factors, obstructing ventricular systolic and/or diastolic function. An epidemiological survey shows that the disease is prone to people over 65 years old, with a high mortality and hospitalization rate, which causes a substantial economic burden. In recent years, modern medicine has made remarkable progress in the prevention and treatment of HF. However, there is still a lack of ideal treatment countermeasures in diuretics resistance, repeated hospitalization of HF, and the decline of quality of life. In the face of these problems, traditional Chinese medicine has a significant advantage and a broad prospect in treating HF due to its multidirectional, multitarget, multimechanism, and small side effects. The occurrence of HF is closely related to ion defects, inflammation and remodelling, myocardial hypertrophy, etc., and the discovery of new biomarkers is beneficial to its early diagnosis [19, 20].

The compensations and characteristics of old Chinese medicine diagnosis and management of HF are obvious, and rich clinical experience has been accumulated. The combination of Chinese and Western medicine allows patients to get comprehensive treatment in all directions [21]. ZWD is a classic Chinese medicine prescription for HF, and it is urgent to study its pharmacological mechanism.

In the current research, 59 dynamic apparatuses of ZWD was found, including kaempferol, paeoniflorin, (+) -catechin, 6-methylgingediacetate 2, and (R) -norcoclaurine. Kaempferol blocks Akt/glycogen kinase (GSK)-3*β*. The apoptosis of the signal transduction pathway of p38 mitogen-activated protein kinase/extracellular signal-regulated kinase can inhibit apoptosis, thus improving the cardiac protection of KF. Kaempferol is a kind of flavonoid compound, which prevents the cardiac remodelling induced by angiotensin II by reducing the inflammation and oxidative stress induced by angiotensin II [22, 23]. Kaempferol significantly inhibited the expression of high glucose-induced inflammatory cytokines and ROS production, thus reducing the fibrosis response and apoptosis in vitro. Kaempferol suppresses nuclear factors- *κ* B (NF- *κ*B) and nuclear translocation, and the protective action of DCM is mediated by nuclear factors-like erythrocytes 2 P45 related factor 2 (Nrf-2). Kaempferol reduced diabetes-induced cardiac fibrosis and apoptosis in type 1 diabetic mice generated by streptozotocin. These alterations were similarly associated with decreased inflammation and oxidative stress in diabetic mice's hearts [24]. Sinapine decreased the activity of the ASK1/JNK1/2/p38 signal pathway and the amplification of H9c2 cardiomyocytes in vitro. Furthermore, in mice, kaempferol was found to protect the heart and H9c2 cells from the effects of oxidative stress [25]. Catechins significantly reduced the expression level of inflammatory cytokines and provided cardiac protection for adriamycin-induced cardiac toxicity through antioxidation and anti-inflammatory properties [26]. Catechins regulate CREB/lncRNA Miat/Akt/Gsk-3*β*. The pathway abridged the programmed cell death of myocardial cells induced by H/R [27]. Paeoniflorin also inhibited collagen deposition in the heart under pressure overload, TGF*β*, CTGF, I*α*, and III*α*. The expression of collagen and phosphorylation of Smad2 and Smad3. Eonoside can inhibit the apoptosis of myocardial cells, and the introduction of AB by the cleavage of Bax and caspase 3 reduces cardiac hypertrophy, fibrosis, apoptosis, and inflammation in stress overload mice and improves left ventricular function [28]. Paeoniflorin's goal is to improve remodelling induced by distress in spontaneously hypertensive rats by modulating the MAPK signalling system [29]. Paeoniflorin can reduce heart remodelling and improve cardiac function, as well as reduce CVF level, collagen volume around blood vessels, and TGF in rats with HF- *β*. The expression of Smad3 and 1 suggests that the potential mechanism of cardiac protection may be related to TGF- *β* 1/the down-down height correlation of the Smad signal path [30].

We also got 83 common target genes of disease drugs. Through PPI network analysis, we got the hub genes with the highest degree: AKT1, IL6, Jun, and MAPK8. AKT1 is a critical gene in the PI3k/Akt signalling pathway. AKT1 is involved in cell functions such as apoptosis, glucose metabolism, and cell proliferation and growth [31]. Akt activity was raised when interacting with actin and HSP90, while it was lowered when interacting with other proteins (such as CTMP and TRB3). The function of Akt in cardiovascular processes such as cell survival, growth, proliferation, angiogenesis, vasodilation, and cell metabolism is determined by its activity in downstream targets. Akt enhances cell survival by activating caspase-9, Yap, Bcl-2, and Bax. Akt mediated eNOS activation, vasodilation, and angiogenesis, as well as increased VEGF release. Through its downstream targets, GSK3 and GLUT4, Akt can boost cell metabolism.

Many cardiovascular diseases, such as atherosclerosis, cardiac hypertrophy, and vascular remodeling, are linked to changes in Akt signal transduction [32]. In the large heterogeneous HF cohort, more than 50 per cent of patients found that IL-6 levels were elevated and were associated with iron deficiency, reduced LVEF, atrial fibrillation, and poor clinical outcomes. These findings require further study of IL-6 as a potential therapeutic target in specific HF subgroups [35]. In HF, there was an inequity between proinflammatory and anti-inflammatory cytokines. The concentration of several interleukins in HF increased, including IL-1*β*, IL-6, IL-8, IL-9, IL-10, IL-13, IL-17, and IL-18 were downregulated, while IL-5, IL-7, or IL-33 were downregulated [34]. The concentration of inflammatory medium is related to cardiac function, which can be an HF marker and a predictor of adverse outcome or mortality. IL-6 may cause myocardial damage and dysfunction in chronic HF syndrome caused by different reasons. As the cause of cardiomyopathy, myocarditis, rejection of allogeneic transplantation, and CHF in the condition of left ventricular assist device (LVAD), the IL-6 level in circulation is related to the severity of left ventricular dysfunction and is also a powerful predictor of subsequent clinical results. In viral myocarditis, continuous and excessive production of IL-6 will destroy the cytokine network and virus clearance, thus promoting myocardial damage. Although IL-6 may be essential in the process of virus antigen presentation, early activation of the immune response and weakening of virus replication seem to be significant in animal models of viral myocarditis. IL-6 can induce cardiac hypertrophy by IL-6 signalling receptor glycoprotein 130 [35].

Response to the drug, lipopolysaccharide, bacterial molecule response, and other BPs; membrane raft, membrane microdomain, plasma membrane raft, caveola, and other MFs; nuclear receptor activity, live activated transcription factor activity, peptide binding, G protein-coupled amine receptor activity, and other MFs. The AGE-RAGE signalling pathway in diabetes composition, fluid shear stress and atherosclerosis, TNF signalling pathway, TB, and Kaposi sarcoma linked herpesvirus infection are all included in the KEGG enrichment study. It is known that the formation and accumulation of advanced glycation end products (ages) are accelerated in diabetes. In addition, even with improved glycemic control, ages hardly degraded and remained in diabetic vessels for a long time. Therefore, aging can explain why previously accumulated diabetic exposure may lead to the current progression of diabetic vascular complications [36]. Kaempferol can alleviate myocardial ischemia-reperfusion injury in diabetic rats by reducing the oxidative stress and inflammatory response induced by AGE-RAGE/MAPK [37]. Endothelial cells convert friction (fluid shear stress) from blood flow into biochemical signals, which influence gene expression and cell activity via specific processes and pathways. These routes improve tissue flow by shaping the circulatory system during development, postpartum, and maturity. Atherosclerosis and vascular abnormalities are also caused by the same way [38]. TNF receptor activation in myocardial ischemia/reperfusion damage and protection has a conflicting function. Excess TNF expression and subsequent activation of TNF receptor type 1 in cardiomyocytes can result in systolic dysfunction, hypertrophy, fibrosis, and cell death.

In contrast, lower levels of TNF can result in systolic dysfunction, hypertrophy, fibrosis, and cell death. In type 2 cardiomyocytes, the presence of TNF and subsequent activation of the TNF receptor produced protective benefits. In addition to its concentration and receptor subtypes, TNF*α* the myocardial effect of CPB also depends on the duration of exposure and its location [39].

Discovery Studio (DS, v2016) is a younger breed of molecular modeling software that uses the method of the CDOCKER module to randomise search the conformational changes of molecules and then optimises each shape in the receptor active site region using the stochastic optimization method [40, 41]. We can simulate the docking of active components and target targets in the component target signalling pathway network diagram using molecular docking technology, and it can help with the discovery of effective ingredients with clinical potential, drug design, and precursor optimization by analysing relevant binding parameters and binding conformation [42]. Several components, including kaempferol, P (+) - catechin, 6-methylgediacetate 2, and (R) - norcoclaurine, have favourable docking conformations. The forces include the Van der Waals force, the Yanqiao force, and the PI alkyl force. Molecular docking proved that ZWD could regulate the PPI network by binding with a hub protein and intervening in HF through different signalling pathways [43].

## 5. Conclusion

Based on the network pharmacology method, 59 components of ZWD and 83 target genes related to HF were obtained. Through the PPI network, four potential therapeutic targets were identified: AKT1, IL6, Jun, and mapk8. The beneficial components of ZWD might intervene HF through the AGE-RAGE signalling pathway in diabetic composition, fluid shear stress and atherosclerosis, the TNF signalling pathway, TB, and Kaposi sarcoma related herpesvirus infection, according to a KEGG enrichment study. Through molecular docking technology, it was found that a variety of effective components in ZWD, such as kaempferol, could combine with hub protein, which preliminarily verified network pharmacology and laid a foundation for subsequent pharmacological research.

## Figures and Tables

**Table 1:** Operative ingredients of ZWD.

Drug	Mol ID	Mol Name	OB	DL
*Paeoniae Radix* Alba	MOL001910	11alpha,12alpha-epoxy-3beta-23-dihydroxy-30-norolean-20-en-28,12beta-olide	64.77	0.38
*Paeoniae Radix* Alba	MOL001918	Paeoniflorgenone	87.59	0.37
*Paeoniae Radix* Alba	MOL001919	(3S,5R,8R,9R,10S,14S)-3,17-Dihydroxy-4,4,8,10,14-pentamethyl-2,3,5,6,7,9-hexahydro-1H-cyclopenta[a]phenanthrene-15,16-dione	43.56	0.53
*Paeoniae Radix* Alba	MOL001921	Lactiflorin	49.12	0.8
*Paeoniae Radix* Alba	MOL001924	Paeoniflorin	53.87	0.79
*Paeoniae Radix* Alba	MOL001925	Paeoniflorin_qt	68.18	0.4
*Paeoniae Radix* Alba	MOL001928	Albiflorin_qt	66.64	0.33
*Paeoniae Radix* Alba	MOL001930	Benzoyl paeoniflorin	31.27	0.75
*Paeoniae Radix* Alba	MOL000211	Mairin	55.38	0.78
*Paeoniae Radix* Alba	MOL000358	Beta-sitosterol	36.91	0.75
*Paeoniae Radix* Alba	MOL000359	Sitosterol	36.91	0.75
*Paeoniae Radix* Alba	MOL000422	Kaempferol	41.88	0.24
*Paeoniae Radix* Alba	MOL000492	(+)-Catechin	54.83	0.24
*Atractylodes macrocephala* Koidz	MOL000020	12-Senecioyl-2E,8E,10E-atractylentriol	62.4	0.22
*Atractylodes macrocephala* Koidz	MOL000021	14-Acetyl-12-senecioyl-2E,8E,10E-atractylentriol	60.31	0.31
*Atractylodes macrocephala* Koidz	MOL000022	14-Acetyl-12-senecioyl-2E,8Z,10E-atractylentriol	63.37	0.3
*Atractylodes macrocephala* Koidz	MOL000028	*α*-Amyrin	39.51	0.76
*Atractylodes macrocephala* Koidz	MOL000033	(3S,8S,9S,10R,13R,14S,17R)-10,13-Dimethyl-17-[(2R,5S)-5-propan-2-yloctan-2-yl]-2,3,4,7,8,9,11,12,14,15,16,17-dodecahydro-1H-cyclopenta[a]phenanthren-3-ol	36.23	0.78

## Data Availability

The data used to support this work are available from the corresponding author on reasonable request.

## Supplementary Materials

Supplementary Materials Appendix: Supplementary materials. Table 2. Operative ingredients of ZWD (Continued).
