# Supplementary material for: Mechanism of Zhen Wu Decoction in the Treatment of Heart Failure Based on Network Pharmacology and Molecular Docking
Source: Evid Based Complement Alternat Med. 2022 Mar 15;2022:4877920. doi: 10.1155/2022/4877920 (PMC8941561; doi:10.1155/2022/4877920)
Supplement: Supplementary Materials — Appendix: Supplementary materials. Table 2. Operative ingredients of ZWD (Continued). [file 4877920.f1.docx]

Appendix: Supplementary materials

Table.2 Operative ingredients of ZWD (Continued)

| Drug | MOL ID | MOL NAME | OB | DL |
| --- | --- | --- | --- | --- |
| *Atractylodes Macrocephala* Koidz | MOL000049 | 3β-acetoxyatractylone | 54.07 | 0.22 |
| *Atractylodes Macrocephala* Koidz | MOL000072 | 8β-ethoxy atractylenolide Ⅲ | 35.95 | 0.21 |
| *Poria Cocos*(Schw.) Wolf | MOL000273 | (2R)-2-[(3S,5R,10S,13R,14R,16R,17R)-3,16-dihydroxy-4,4,10,13,14-pentamethyl-2,3,5,6,12,15,16,17-octahydro-1H-cyclopenta[a]phenanthren-17-yl]-6-methylhept-5-enoic acid | 30.93 | 0.81 |
| *Poria Cocos*(Schw.) Wolf | MOL000275 | trametenolic acid | 38.71 | 0.8 |
| *Poria Cocos*(Schw.) Wolf | MOL000276 | 7,9(11)-dehydropachymic acid | 35.11 | 0.81 |
| *Poria Cocos*(Schw.) Wolf | MOL000279 | Cerevisterol | 37.96 | 0.77 |
| *Poria Cocos*(Schw.) Wolf | MOL000280 | (2R)-2-[(3S,5R,10S,13R,14R,16R,17R)-3,16-dihydroxy-4,4,10,13,14-pentamethyl-2,3,5,6,12,15,16,17-octahydro-1H-cyclopenta[a]phenanthren-17-yl]-5-isopropyl-hex-5-enoic acid | 31.07 | 0.82 |
| *Poria Cocos*(Schw.) Wolf | MOL000282 | ergosta-7,22E-dien-3beta-ol | 43.51 | 0.72 |
| *Poria Cocos*(Schw.) Wolf | MOL000283 | Ergosterol peroxide | 40.36 | 0.81 |
| *Poria Cocos*(Schw.) Wolf | MOL000285 | (2R)-2-[(5R,10S,13R,14R,16R,17R)-16-hydroxy-3-keto-4,4,10,13,14-pentamethyl-1,2,5,6,12,15,16,17-octahydrocyclopenta[a]phenanthren-17-yl]-5-isopropyl-hex-5-enoic acid | 38.26 | 0.82 |
| *Poria Cocos*(Schw.) Wolf | MOL000287 | 3beta-Hydroxy-24-methylene-8-lanostene-21-oic acid | 38.7 | 0.81 |
| *Poria Cocos*(Schw.) Wolf | MOL000289 | pachymic acid | 33.63 | 0.81 |
| *Poria Cocos*(Schw.) Wolf | MOL000290 | Poricoic acid A | 30.61 | 0.76 |
| *Poria Cocos*(Schw.) Wolf | MOL000291 | Poricoic acid B | 30.52 | 0.75 |
| *Poria Cocos*(Schw.) Wolf | MOL000292 | poricoic acid C | 38.15 | 0.75 |
| *Poria Cocos*(Schw.) Wolf | MOL000296 | hederagenin | 36.91 | 0.75 |
| *Poria Cocos*(Schw.) Wolf | MOL000300 | dehydroeburicoic acid | 44.17 | 0.83 |
| *Aconiti Lateralis* Radix | MOL002211 | 11,14-eicosadienoic acid | 39.99 | 0.2 |
| *Aconiti Lateralis* Radix | MOL002388 | Delphin_qt | 57.76 | 0.28 |
| *Aconiti Lateralis* Radix | MOL002392 | Deltoin | 46.69 | 0.37 |
| *Aconiti Lateralis* Radix | MOL002393 | Demethyldelavaine A | 34.52 | 0.18 |
| *Aconiti Lateralis* Radix | MOL002394 | Demethyldelavaine B | 34.52 | 0.18 |
| *Aconiti Lateralis* Radix | MOL002395 | Deoxyandrographolide | 56.3 | 0.31 |
| *Aconiti Lateralis* Radix | MOL002397 | karakoline | 51.73 | 0.73 |
| *Aconiti Lateralis* Radix | MOL002398 | Karanjin | 69.56 | 0.34 |
| *Aconiti Lateralis* Radix | MOL002401 | Neokadsuranic acid B | 43.1 | 0.85 |
| *Aconiti Lateralis* Radix | MOL002406 | 2,7-Dideacetyl-2,7-dibenzoyl-taxayunnanine F | 39.43 | 0.38 |
| *Aconiti Lateralis* Radix | MOL002410 | benzoylnapelline | 34.06 | 0.53 |
| *Aconiti Lateralis* Radix | MOL002415 | 6-Demethyldesoline | 51.87 | 0.66 |
| *Aconiti Lateralis* Radix | MOL002416 | deoxyaconitine | 30.96 | 0.24 |
| *Aconiti Lateralis* Radix | MOL002419 | (R)-Norcoclaurine | 82.54 | 0.21 |
| *Aconiti Lateralis* Radix | MOL002421 | ignavine | 84.08 | 0.25 |
| *Aconiti Lateralis* Radix | MOL002422 | isotalatizidine | 50.82 | 0.73 |
| *Aconiti Lateralis* Radix | MOL002423 | jesaconitine | 33.41 | 0.19 |
| *Aconiti Lateralis* Radix | MOL002433 | (3R,8S,9R,10R,13R,14S,17R)-3-hydroxy-4,4,9,13,14-pentamethyl-17-[(E,2R)-6-methyl-7-[(2R,3R,4S,5S,6R)-3,4,5-trihydroxy-6-[[(2R,3R,4S,5S,6R)-3,4,5-trihydroxy-6-(hydroxymethyl)oxan-2-yl]oxymethyl]oxan-2-yl]oxyhept-5-en-2-yl]-1,2,3,7,8,10,12,15,16,17-decahydr | 41.52 | 0.22 |
| *Aconiti Lateralis* Radix | MOL002434 | Carnosifloside I_qt | 38.16 | 0.8 |
| *Aconiti Lateralis* Radix | MOL000359 | sitosterol | 36.91 | 0.75 |
| *Aconiti Lateralis* Radix | MOL000538 | hypaconitine | 31.39 | 0.26 |
| *Zingiber Officinale* Roscoe | MOL000358 | beta-sitosterol | 36.91 | 0.75 |
| *Zingiber Officinale* Roscoe | MOL006129 | 6-methylgingediacetate2 | 48.73 | 0.32 |
| *Zingiber Officinale* Roscoe | MOL000449 | Stigmasterol | 43.83 | 0.76 |
| *Zingiber Officinale* Roscoe | MOL001771 | poriferast-5-en-3beta-ol | 36.91 | 0.75 |
| *Zingiber Officinale* Roscoe | MOL008698 | Dihydrocapsaicin | 47.07 | 0.19 |
